# The key role of major and trace elements in the formation of five common urinary stones

**DOI:** 10.1186/s12894-024-01498-5

**Published:** 2024-05-30

**Authors:** Yu Tian, Guilin Han, Shudong Zhang, Ziyang Ding, Rui Qu

**Affiliations:** 1 Department of Urology, Peking University Third Hospital, Beijing, 100191 China; 2 Institute of Earth Sciences, China University of Geosciences, No. 29 Xueyuan Road, Haidian District, Beijing, 100083 China; 3 Frontiers Science Center for Deep-time Digital Earth, Institute of Earth Sciences, China University of Geosciences, Beijing, 100083 China

**Keywords:** Urinary stones, Trace element, Elemental composition, Principal component analysis

## Abstract

**Background:**

Urolithiasis has emerged as a global affliction, recognized as one of the most excruciating medical issues. The elemental composition of stones provides crucial information, aiding in understanding the causes, mechanisms, and individual variations in stone formation. By understanding the interactions between elements in various types of stones and exploring the key role of elements in stone formation, insights are provided for the prevention and treatment of urinary stone disease.

**Methods:**

This study collected urinary stone samples from 80 patients in Beijing. The chemical compositions of urinary stones were identified using an infrared spectrometer. The concentrations of major and trace elements in the urinary stones were determined using Inductively Coupled Plasma Optical Emission Spectrometry (ICP-OES) and Inductively Coupled Plasma Mass Spectrometry (ICP-MS), respectively. The data were processed using correlation analysis and Principal Component Analysis (PCA) methods.

**Results:**

Urinary stones are categorized into five types: the calcium oxalate (CO) stone, carbonate apatite (CA) stone, uric acid (UA) stone, mixed CO and CA stone, and mixed CO and UA stone. Ca is the predominant element, with an average content ranging from 2.64 to 27.68% across the five stone groups. Based on geochemical analysis, the high-content elements follow this order: Ca > Mg > Na > K > Zn > Sr. Correlation analysis and PCA suggested significant variations in the interactions between elements for different types of urinary stones. Trace elements with charges and ionic structures similar to Ca may substitute for Ca during the process of stone formation, such as Sr and Pb affecting the Ca in most stone types except mixed stone types. Moreover, the Mg, Zn and Ba can substitute for Ca in the mixed stone types, showing element behavior dependents on the stone types.

**Conclusion:**

This study primarily reveals distinct elemental features associated with five types of urinary stones. Additionally, the analysis of these elements indicates that substitutions of trace elements with charges and ion structures similar to Ca (such as Sr and Pb) impact most stone types. This suggests a dependence of stone composition on elemental behavior. The findings of this study will enhance our ability to address the challenges posed by urinary stones to global health and improve the precision of interventions for individuals with different stone compositions.

**Supplementary Information:**

The online version contains supplementary material available at 10.1186/s12894-024-01498-5.

## Introduction

Urolithiasis stands out as a prevalent and distressing medical issue experienced globally [1, 2]. It has the potential to lead to the deterioration of renal function and permanent damage to the urinary system [3, 4]. Urolithiasis impacts 5–15% of the population in industrialized countries [5]. In the Western world, the lifetime risk of developing symptoms of urolithiasis ranges from 10 to 15%, and in the Middle East, the risk can be as high as 25% [3, 4]. Urolithiasis affects 5 − 15% of the population in industrialized countries [5]. In the Western world, the lifetime risk of experiencing symptoms related to urolithiasis ranges from 10 to 15%, and in the Middle East, this risk can escalate to as high as 25% [6]. According to a previous study in 2008 on the prevalence of urinary stone disease in the Chinese population reported an overall prevalence of 4.0%, with 4.8% for men and 3.0% for women [7]. By 2013 a statistic showed that the prevalence of urinary stone disease in China was 5.8% for urinary stones, 6.5% for men, and 5.1% for women. The incidence is clearly on the increase [8]. It is noteworthy that urinary stones often exhibit frequent recurrence. Without proper preventive measures, the recurrence rates within 1 year and 10 years after stone removal can exceed 10% and 50%, respectively [9]. A recent report from Iceland indicates that the recurrence rate of pediatric stones after removal ranges from 26% at 5 years post-surgery to 46% at 20 years post-surgery [10]. The increasing incidence and recurrence rates underscore the significant societal medical challenge posed by urinary stones [11, 12].

Despite significant progress in comprehending the multifactorial pathophysiology of urinary stone formation, a comprehensive and satisfactory explanation of this process remains elusive [6, 13]. Early investigations into urinary stone disease have provided insights into the composition, mineralogy, structure, formation processes, and geo-environmental factors [14, 15]. Recently, there has been a growing focus on the role of the major and trace elements in lithogenesis [16, 17]. Crucial for optimal development and metabolic functioning in all organisms, many researchers contend that trace elements play a pivotal role in mediating the onset and progression of various diseases [18, 19]. Certain trace elements are well documented to influence the crystallization of urinary stone components by acting on the surface of the crystals [6, 20]. Moreover, they may exert influence on the external morphology of crystal growth, either accelerating or decelerating the crystallization process [6, 18]. Both the major and trace elements are inherent in the human body, playing a vital role in individual health [2]. Numerous trace elements are indispensable for specific metabolic processes, undergoing temporary storage before being excreted through the urinary system to maintain overall physiological balance [21].

Urinary stones comprise approximately 40 components [22], with the primary chemical constituents of stones being CO, calcium phosphate (hydroxyapatite and CA), UA, Cystine and struvite [23, 24]. CO emerges as the most prevalent mineral phase in uroliths, occurring with a frequency of around 70-75% [25]. Calcium phosphate stones are divided into hydroxyapatite and CA, and their lithogenic effect depends on urinary pH and Ca concentration. Considering the reduced solubility of calcium phosphate in alkaline conditions, excess excretion of urinary Ca combined with alkaline urine (pH > 6.0) favors the formation of calcium phosphate stones [26]. The prevalence of UA stones worldwide is approximately 10–15%. Risk factors for UA stone formation include conditions associated with elevated urinary UA concentration, low urine volume, and low urinary pH [27]. Struvite stones, commonly referred to as infectious stones, appear to be associated with urinary tract infections. The infectious stones involved the bacteria activities that enhance or inhibit the crystallization process like nucleation and crystal growth [28, 29]. Elevated cystine concentration can lead to cystine precipitation and the formation of cystine stones, with a prevalence of less than 1% worldwide [26]. The vast majority of urinary stones contain two or more components, with various combinations. A study showed that urinary stones are mainly composed of two components (50.9%), followed by single-component stones (27.1%) and three-component stones (21.9%) [30]. Microscopically, mixed-type stones exhibit a greater variety of crystal growth patterns, with more irregular and diverse shapes and structures [31]. The formation of urinary stones typically initiates with an elevation in the concentration of certain solutes in urine, leading to a state of supersaturation [32]. The solutes in this supersaturated urine gradually crystallize, forming minute particles that progressively grow into solid cores. These cores aggregate over time, culminating in the development of complete urinary stones, which may accumulate within the urinary stones and eventually be expelled through the urethra, causing symptoms of urinary tract stones [33]. Despite an incomplete understanding of the stone precipitation process, urinary stone analysis plays a crucial role in identifying the potential etiology and pathophysiology of stone formation. This, in turn, can contribute to efforts to prevent the formation of urinary stones [34, 35].

Analyzing the elements in urinary stones is crucial for studying urinary stone formation. However, there have been few comparative studies on the elemental behaviors of different types of urinary stones. This study aims to explore the elemental characteristics of five common types of urinary stones through correlation analysis and PCA. We hope to provide reference data for the treatment and prevention of urinary stone diseases.

## Materials and methods

Urinary stone samples from 80 patients were collected during urinary stone removal surgeries at the Peking University Third Hospital. Before the experiment, the Peking University Third Hospital Medical Science Research Ethics Committee approved this study (protocol code (2021) MSREC 475-1). This study involves only data collection and analysis, which does not pose risks to patient safety and safeguards patients’ personal information (only age and gender are disclosed). Therefore, patients are not required to sign an informed consent form. The application for waiver of patient informed consent is included in the ethics review approval notification. All experimental processes, including cleaning and dilution, were performed using ultrapure water (18.25 MΩ.cm, Cascada™, Kirkland, Washington, USA). All urinary stone samples were subjected to multiple washes with ultrapure water and ultrasonic treatment in a water bath for 30 min to remove urine, blood fragments, and organic residues. Subsequently, the samples were transferred to small dried bottles and air-dried with sterile gauze. The dried urinary stone samples were ground into powder using sterile agate mortars and pestles. Mix 1 mg of stone powder with 200 mg of anhydrous potassium bromide. Subsequently, the mixture is placed in an oven and dried at 70–100 °C, then removed and pressed into semi-transparent thin sheets. Quickly scan these sheets using an infrared spectrometer (LIIR-20, Lambda Scientific, Tianjin, China) with a frequency range from 4000 to 400 cm^− 1^ and a resolution of 4 cm^− 1^ [36, 37]. Finally, the computer plotted the spectrum and automatically analyzed the composition of the stones (Software: LIIR 7.2.1.50u, Lambda Scientific). According to previous research, the digestion method for urinary stones involved HF-HNO_3_-HClO_4_-HCl [17, 38]. In polytetrafluoroethylene (PFA) crucibles containing the samples, 3 ml of concentrated HF, 1 ml of concentrated HNO_3_, and 0.5 ml of HClO_4_ were added and heated at 120 °C for 48 h. The solution was subsequently evaporated, and 1 ml of concentrated HNO_3_ and 3 ml of concentrated HCl (aqua regia) were introduced to the PFA crucibles, followed by heating at 120 °C for 48 h [38]. Prior to measurement, all solutions underwent final drying and redissolving to a constant volume. The digested samples underwent analysis for the major and trace elements at the Institute of Geographic Sciences and Natural Resources Research, Chinese Academy of Sciences. This analysis employed ICP-OES (Optima 5300DV, PerkinElmer, Waltham, MA, USA) and ICP-MS (Elan DRC-e, Perkin Elmer, Waltham, MA, USA). Ca, Mg, Na, and K were determined by ICP-OES, while Sr, Zn, Li, Ti, Cu, Se, Rb, Ba, and Pb were determined by ICP-MS. Quality control for the method was ensured by using blank samples, replicate samples, and standard reference materials (Alfa 046318 and Alfa 036371), with an analysis accuracy greater than ± 5%.

PCA was conducted using IBM SPSS version 22.0. T-tests were employed to compare measurement data between different groups. Pearson correlation was utilized to analyze the relationships between elements in various types of urinary stones (Origin 2021). The significance level was defined as *p* < 0.05 for statistical significance and *p* < 0.01 for highly significant results.

## Results and discussion

Infrared spectroscopy proves to be a convenient method for analyzing the mineral composition of urinary stones [39]. Using the mineral composition obtained through infrared spectroscopy diffraction, urinary stones can be classified into four distinct mineral groups, including calcium oxalate monohydrate (COM, CaC_2_O_4_·H_2_O), also known as Whewellite, calcium oxalate dihydrate (COD, CaC_2_O_4_·_2_H_2_O), also known as Weddellite, CA (Ca_10_(PO_4_)_6_CO_3_·H_2_O), and UA (C_5_H_4_N_4_O_3_) [40, 41]. Other types of stones were not collected or were too few in number to be considered in this study (such as hydroxyapatite, struvite stones, etc.). Previous research has demonstrated that Whewellite stones and Weddellite stones are distinct minerals with significant differences in crystal structure [16]. Whewellite crystals exhibit a flat monoclinic prismatic shape, displaying a radial pattern and concentric layers, Weddellite crystals have a shape resembling an eight-faced bipyramid [31, 42]. The variations in crystal structure are related to the adhesive characteristics of their crystal faces [42]. However, due to their high Ca content, they are collectively referred to as calcium oxalate stones. We only collected Whewellite stones and mixed stones containing both Whewellite and Weddellite. Existing studies have demonstrated that the elemental behavior of these stone types is similar [43, 44]. Therefore, we categorize Whewellite stones and mixed stones of Whewellite and Weddellite under the group of CO stones. Representative infrared spectra for this study are shown in Fig S1 of the Supplementary file. In the analysis of the 80 stones examined in this study, due to the relative rarity of stone types other than calcium oxalate stones, we incorporated data from 11 stones in our previous research [17, 41], enhancing the statistical significance of the analytical results, 50 were classified as the CO stone group, including COM and COD. 9 were classified as the CA stone group, 8 as the UA stone group, 5 as the mixed CO and CA stone group, and 8 stones were classified as the mixed CO and UA stone group. Among all samples, the proportion of urinary stones in males reaches 80%. The average age of patients with stones was 55.38. There are only 13 cases of urinary stones in individuals below the age of 40, while the highest number of stone cases, 20 in total, occurs in the age group of 51–60 years.

Urinary stones typically consist of organic and inorganic substances, which can potentially jeopardize the health of biological organs. Identifying the elemental composition of urinary stones can provide valuable information for implementing alleviative measures in the treatment of patients [45]. Table 1 presents the results of the major and trace metal content along with their average values and concentrations for each mineral group of urinary stones. Ca^2+^ constitutes the primary component of all types of urinary stones, and the detailed data are presented in Table S1. The average concentration of Ca^2+^ in the CO stone group (27.68%) exceeds that in the CA stone group (18.95%), being the lowest in the UA stone group with an average concentration of 2.64%. It should be noted that the Ca^2+^ content is influenced by factors such as the types of food and beverages consumed by patients, including dairy products, eggs, tea, and hard water, or pathological conditions such as idiopathic hypercalciuria, absorptive or resorptive hypercalciuria [46, 47]. Mg is an essential element in biomineralization [48], and its detection in urinary stones frequently serves as an indicator of elevated concentration in the body [18]. The average concentration of Mg^2+^ in the CA stone group is significantly higher than in other stone groups, measuring 38.24 mg/g. Typically, higher levels of Mg are observed in struvite stones and struvite-calcium phosphate mixed stones [49]. However, relevant studies have also reported elevated Mg content in pure calcium phosphate stones [49]. This could be attributed to the crystallization process during the formation of CA stones, where minerals in the urine can combine to form crystals. Mg commonly replaces a portion of Ca, contributing to the crystal structure of the stones along with phosphate and carbonate ions [50]. Due to their porous nature, CA stones can absorb various chemical elements. Yet, the precise role of Mg in urinary stone formation remains not fully understood [51]. Dietary sources and hard water, along with some medications, contribute to variations in Mg content [24]. The CA stones may exhibit a stronger capacity for absorbing alkali metals (Na, K). The average concentrations of Na and K in the CA stones are 7527.38 µg/g and 1218.02 µg/g, respectively. Similarly, elevated levels are observed in the CO stones and the mixed CO and CA stones. Conversely, in the UA stones, the concentrations of Na and K are the lowest, measuring 689.33 µg/g and 158.25 µg/g, respectively. The alkali metal content in the mixed CO and UA stones is also significantly reduced, influenced by the presence of the UA stones. These findings suggest that the CA stones may possess distinctive biochemical characteristics in their interactions with alkali metals. The content of trace elements Zn and Sr exhibits similar characteristics to alkali metals. In the CA stones, their concentrations are relatively high at 1210.35 µg/g and 266.14 µg/g, respectively. Conversely, in the UA stones, their concentrations are lower at 14.26 µg/g and 8.72 µg/g, respectively. In addition, other study has also demonstrated that the elemental composition of UA stones exhibits the presence of various trace elements in small quantities [52]. These elemental characteristics may also be reflected in the microscopic structure of the stones, with the microscopic morphology of UA stones showing lower absorption signals and porosity compared to other types of stones [31]. However, in the CO and CA mixed stones, the influence of the CA stones on the content of Zn and Sr is minimal. In the CO and UA mixed stones, their content is significantly affected by the presence of the UA stones. Pb is a potential toxic element that can cause urinary damage at both low and high concentrations, hindering waste elimination from the body [17]. Notably, our study found an average Pb content of 5892.93 µg/kg in the CO stones, indicating no Pb pollution compared to other regions [53]. Furthermore, the content of other elements not mentioned in this study was also found to be low.

**Table 1:** Statistical results of the major and trace elements in different urinary stones

Urinary Stone Type	Parameters	Ca (%)	Mg (mg/g)	Na (µg/g)	K (µg/g)	Sr (µg/g)	Zn (µg/g)	Li (µg/kg)	Ti (µg/kg)	Cu (µg/kg)	Se (µg/kg)	Rb (µg/kg)	Ba (µg/kg)	Pb (µg/kg)
CO (*n* = 50)	Min	22.70	0.17	881.79	1.50	44.86	16.98	0.84	524.87	127.61	31.59	35.80	51.38	916.81
	Max	32.58	29.62	9444.50	2687.67	401.64	1633.34	21.87	38962.76	8733.04	2413.56	2979.65	16385.29	45699.49
	Mean	27.68	2.21	3281.86	363.41	135.86	397.67	4.17	5090.40	801.57	232.01	276.62	3191.36	7124.82
CA (*n* = 9)	Min	10.40	0.52	727.80	129.13	84.24	285.15	1.75	1397.00	144.54	18.80	76.58	681.01	899.24
	Max	27.40	60.20	13554.20	1857.09	486.80	5168.00	1093.30	7282.22	5731.02	235.04	7922.50	38978.00	30673.00
	Mean	18.95	38.24	7527.38	1218.02	266.14	1210.35	282.00	4042.43	1354.80	72.64	4554.07	7925.14	6760.44
UA (*n* = 8)	Min	0.07	0.01	414.61	14.90	1.30	0.90	1.32	19.74	850.00	147.70	251.31	1.54	33.70
	Max	7.16	0.20	833.55	258.47	22.06	74.97	2.54	2662.07	5031.27	637.20	3243.00	1157.01	6537.03
	Mean	2.64	0.07	689.33	158.25	8.72	14.26	2.06	917.68	2271.18	374.11	929.11	286.79	1143.19
Mixed stone (CA and CO (*n* = 5)	Min	25.36	0.29	1275.38	93.95	46.70	12.30	0.10	622.00	278.70	98.40	74.80	473.73	672.10
	Max	29.89	3.03	6369.68	2978.00	261.68	729.27	451.20	9920.00	8595.00	1438.00	1712.00	6465.94	20458.00
	Mean	27.05	1.53	3480.19	1189.51	135.33	302.13	113.91	5905.22	1997.52	438.98	487.66	3274.37	7435.65
Mixed stone CO and UA (*n* = 8)	Min	6.80	0.06	724.33	61.90	15.65	3.60	1.38	42.40	954.47	224.89	143.60	28.33	420.00
	Max	23.88	0.46	1737.32	259.78	133.00	50.64	3.03	759.92	2355.01	549.93	369.95	792.94	15975.00
	Mean	12.75	0.18	1030.04	161.54	43.23	17.39	1.97	311.01	1831.82	370.50	245.42	246.64	2626.25

Note: CO = calcium oxalate, CA = calcium apatite, UA = uric acid

**Fig. 1:**
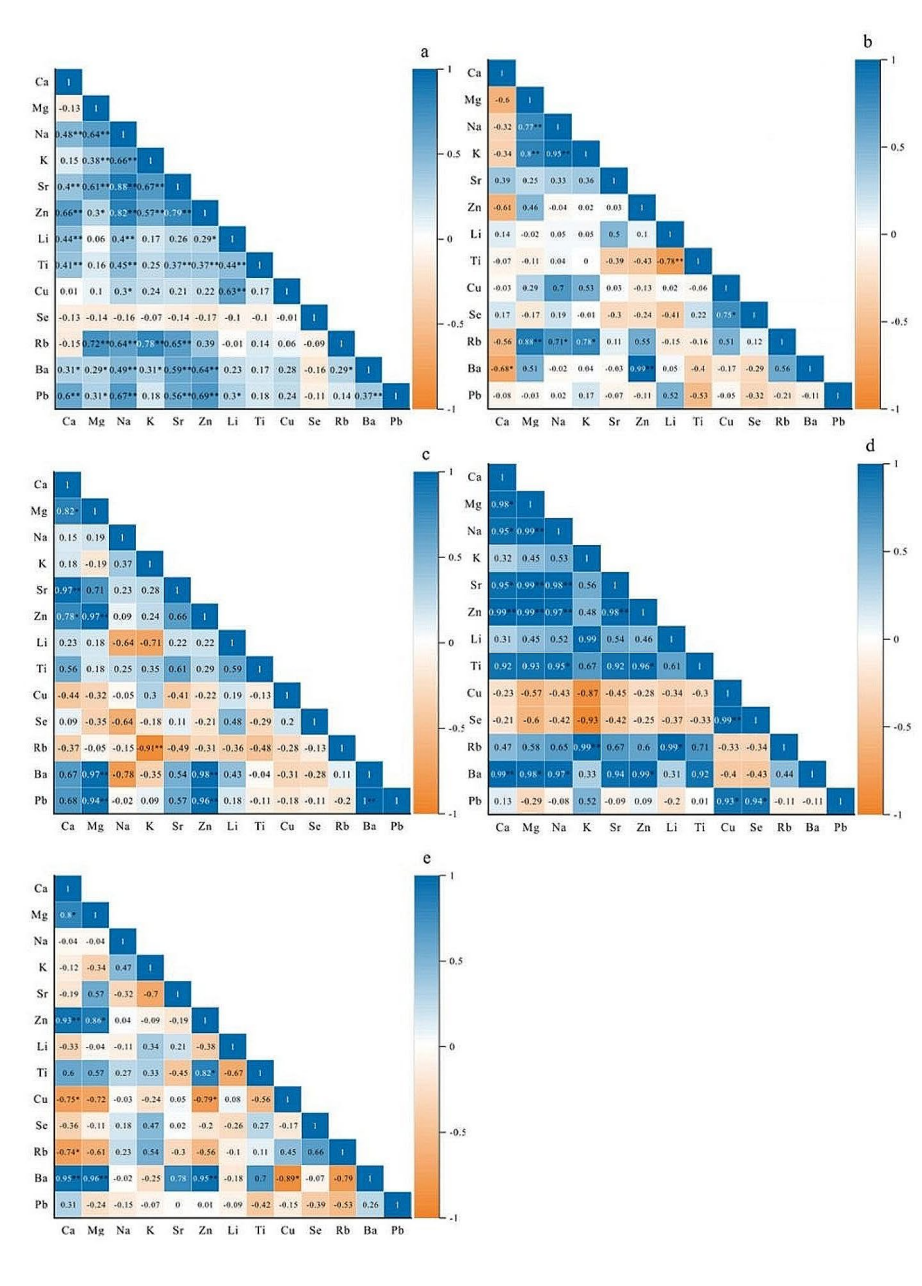
Correlation analysis of the chemical components of urinary stones. **a**. CO; **b**. CA; **c**).UA; **d**. CO + CA; **e**. CO + UA; ** *p* < 0.01; * *p* < 0.05

In addition to identifying the chemical composition and elements in urinary stones and their potential roles in aggregate formation, assessing the correlations between different elements becomes essential to understand the process of stone formation in the urinary tract [45]. To investigate the associations between the major and trace elements in urinary stones, Pearson correlation analyses were performed for each type of urinary stone (Fig. 1). Figure 1a and e presents the result of the correlation analysis for the CO, CA, UA, mixed CO and CA, and the mixed CO and UA stone groups, respectively. In the case of the CO stone group, Ca and Na exhibit a positive correlation (*r* = 0.48), likely attributed to their closely matched ionic radius (116 pm for Ca and 144 pm for Na) [54], This similarity enables Na to substitute for Ca in various rock-forming minerals, such as plagioclase feldspar (sodium-calcium feldspar series NaAl3Si3O8-CaAl2Si2O8) and pyroxene [55, 56]. Ca shows a strong positive correlation with Zn (*r* = 0.66) in the CO stone group, suggesting that these elements may undergo thermodynamically favorable substitution processes or are easily absorbed into the oxalate crystal structure [41]. In the CO stone group, Mg exhibited positive correlations with Na (*r* = 0.64), Sr (*r* = 0.61), and Rb (*r* = 0.72); In the UA stone group, significant positive correlations were observed with Ba (*r* = 0.97), Zn (*r* = 0.97) and Pb(*r* = 0.94); Similarly, in the mixed CO and CA stone group, Mg showed significant positive correlations with Na (*r* = 0.99), Sr (*r* = 0.99), Zn (*r* = 0.99), and Ba (*r* = 0.98). In the mixed CO and UA stone group, Mg demonstrated significant positive correlations with Zn (*r* = 0.86) and Ba (*r* = 0.96). These positive associations with Mg can be explained by its involvement in several crucial processes in the human body, such as contributing to the synthesis and metabolism of proteins and nucleic acids [43]. Mg also serves as a cofactor in many enzyme-catalyzed reactions [16]. When it comes to ion binding, Mg^2+^ exhibits a greater affinity for oxalate than Ca^2+^ because of its smaller size. Early studies indicate that urinary stones exhibit reduced crystallization and growth under high concentrations of Mg^2+^ [52]. This is attributed to Mg’s ability to easily bind to specific sites, thereby lowering the formation rate of oxalates [57]. Furthermore, the positive correlation between Ca and Sr in the CO (*r* = 0.4) stone group suggests that the human body processes Sr like Ca, aiding in the substitution of Sr for Ca in biomineralization processes [58, 59]. Sr and Zn display a positive correlation in the CO stone group (*r* = 0.79), and the mixed CO and CA stone group (*r* = 0.98). Overall, the correlations between elements in the CA stone group and the mixed CO and UA stone group were not significant, while the correlations of Li, Cu, Se, and Pb with other elements were not significant in the UA stone group and the mixed CO and CA stone group, and the overall correlations between other elements were significant in all stone types. The occurrence of urinary stones is believed to be associated not only with geographic distribution, geological environments, and occupational factors but also, more widely, with the suspected influence of various nephrotoxic elements present in drinking water over prolonged periods [60–62]. Due to varying research conclusions, the role of hard water or soft water in urinary stone formation has been a topic of debate [63]. Water hardness is typically determined by the Ca and Mg content in water, with hard water generally containing higher levels of Ca and Mg. A review has concluded that hard water is more conducive to the formation of calcium stones [64]. However, the results of a review survey indicate that 41% of studies suggest using high-Ca hard water to reduce the risk of urinary stone formation [64]. Hard water leads to hypercalciuria, but due to other factors influencing stone formation, the overall impact appears to be a reduction in urinary stone formation [64, 65]. Therefore, hard water does not necessarily promote stone formation; this depends on the type of stones and unique patient factors. In addition, the recurrence frequency of urinary stones also influences their elemental composition. A study on recurrent stones suggests that the distribution of calcium is not uniform, and there are significant concentration differences [66].

**Fig. 2:**
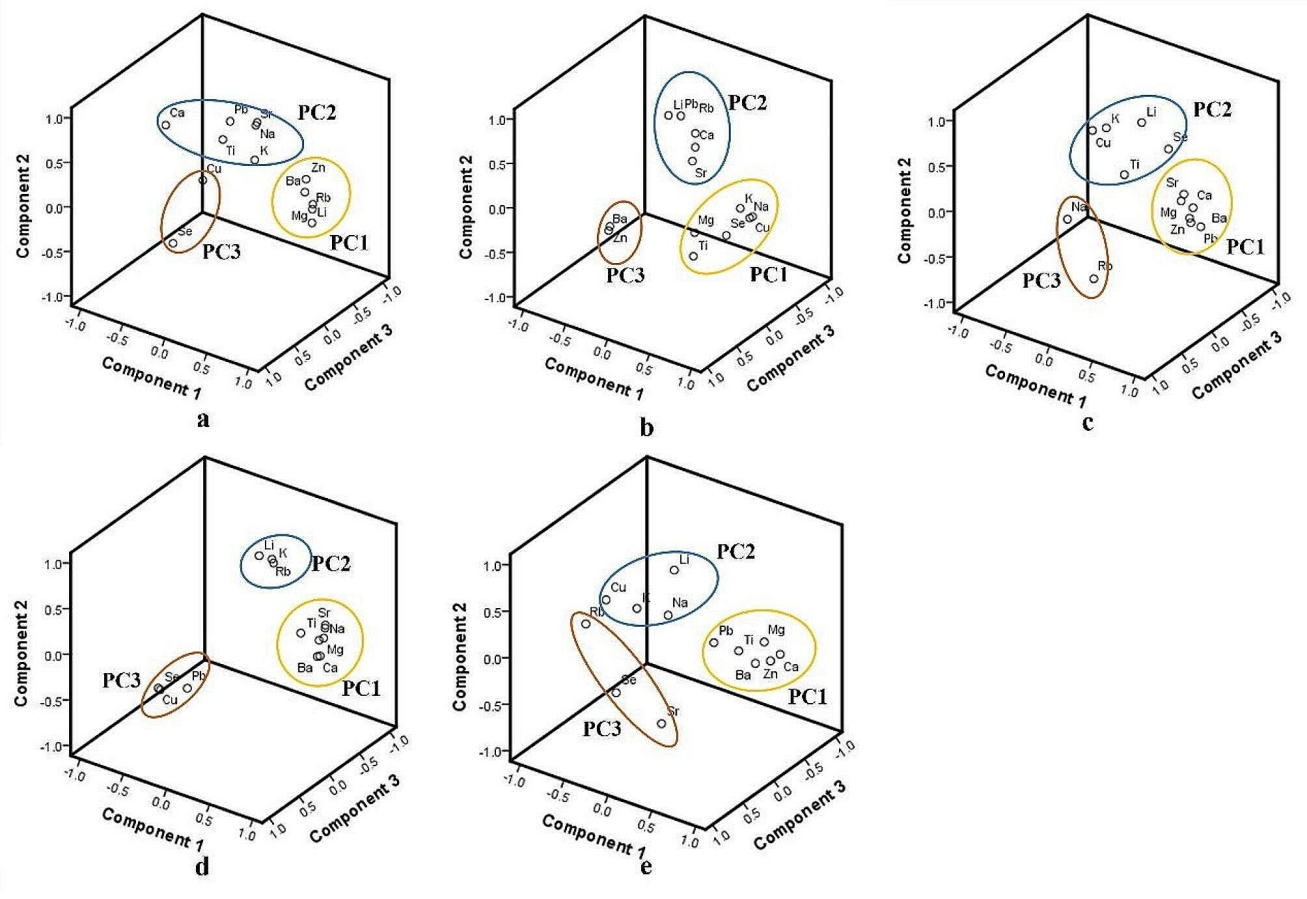
PCA of the chemical components of urinary stones. **a**. CO; **b**. CA; **c**. UA; **d**. CO + CA; **e**. CO + UA

PCA is a common multivariate statistical analysis method that can be used to reduce dimensionality and analyze relationships among various elements. PCA is used to elucidate the relationships among the major elements and to identify potential combinations of the major and trace elements that can play significant roles in the formation of urinary stones. PCA is conducted with the extraction of the maximum rotated variance. PCA was conducted on five categories of urinary stones in this study (Fig. 2). The relationships between the elements in the CO stone group are illustrated in Fig. 2a. Extracting the three highest eigenvalues as PCs account for 74.58% of the total variance, with the first principal component (PC1) explaining 40.42% of the total variance. Figure 2a displays the major loadings in PC1, which include Mg Zn, Li, Rb, and Ba. The second principal component (PC2) contributes to approximately 26.42% of the total variance, with its major loadings being Ca, Na, K, Sr, Ti, and Pb. The third principal component (PC3) is formed by Cu and Se, contributing to 7.74% of the total variance. Certainly, PCA is also being conducted for the CA stone group (Fig. 2b), the UA stone group (Fig. 2c), the mixed CO and CA stone group (Fig. 2d), and the mixed CO and UA stone group (Fig. 2e). For the CA stone group, the three PCs with the highest eigenvalues were extracted, accounting for 73.28% of the total variance. PC1, PC2, and PC3 explain 25.12%, 24.21%, and 23.95% of the total variance, respectively. The primary loadings for PC1 include Mg, Na, K, Ti, Cu, and Se; for PC2, they are Ca, Sr, Li, Rb, and Pb; and for PC3, they are Zn and Ba. For the UA stone group, the three PCs with the highest eigenvalues have been extracted, representing 79.95% of the total variance. PC1, PC2, and PC3 explain 40.02%, 25.51%, and 14.42% of the total variance, respectively. The major loadings for PC1 include Ca, Mg, Sr, Zn, Ba, and Pb; for PC2, they are K, Li, Ti, Cu, and Se; and for PC3, they are Na and Rb. For the mixed CO and CA stone group, the three PCs with the highest eigenvalues account for 99.69% of the total variance. PC1, PC2, and PC3 explain 50.87%, 25.33%, and 23.49% of the total variance, respectively. The major loadings for PC1 include Ca, Mg, Na, Sr, Zn, Ba, and Ti; for PC2, they are K, Li, and Rb; and for PC3, they are Cu, Se, and Pb. For the mixed CO and UA stone group, the three PCs with the highest eigenvalues account for 80.54% of the total variance. PC1, PC2, and PC3 explain 40.24%, 20.24%, and 20.06% of the total variance, respectively. The major loadings for PC1 include Ca, Mg, Zn, Pb, Ba, and Ti; for PC2, they are Na, K, Li, and Cu; and for PC3, they are Sr, Se, and Rb.

The PCA of five types of urinary stones suggests an association between Ca and Sr in four stone categories, excluding the Mixed CO and UA Stone Group. However, the mechanisms by which strontium interacts with calcium in four types of urinary stones still require further investigation. Associations between Pb and Ca were observed in the CO stone group, CA stone group, UA stone group, and the Mixed CO and UA stone group. In the UA stone group and two types of mixed stone group, a potential connection between Ca and trace elements Mg, Zn, and Ba was observed. However, this relationship was not observed in the CO stone group, suggesting that the association between Ca and Mg, Zn, and Ba in the Mixed CO and UA stone group may be predominantly driven by the influence of the UA stone group. Importantly, existing research has indicated that Mg, Zn, Ba, and Sr can substitute for Ca in the formation of urinary stones [49, 67], but the conclusion lacks clarity regarding the differentiation of stone types. In conclusion, the three PCs of the major and trace elements in urinary stones can be explained by potential favorable alternative pathways or preferences in the intake process within the human body, revealing co-precipitation or substitution during the formation of urinary stones. Furthermore, variations in elemental distribution between different types of stone can serve as valuable indicators to tailor nutrient intake based on the specific disease. In future research, we plan to conduct quantitative analysis of mixed urinary stones using advanced techniques such as dual-energy CT and X-ray dark-field tomography. This aims to provide a more comprehensive understanding of the elemental composition and structural characteristics of mixed stones. Similarly, physiological factors influencing the elemental composition of stones may include stone recurrence, metabolic functions in the human body, and the impact of other diseases. These areas are also worthy of in-depth research. This approach will contribute to filling gaps in current research and offer additional insights into the mechanisms of stone formation for further investigation.

## Conclusions

This study investigated the major and trace elements of urinary stones based on different types (CO, CA, and UA, and their mixed types) in Beijing, China. The predominant type of urinary stones is CO, accounting for up to 62.5% of the total, and Ca is the primary element in all types of urinary stones. The metal ions, substitute for Ca in the crystal lattice, are significantly different based on stone types. Specifically, Sr, as a stone-inhibiting element, exerts an influence on Ca in the CO stone group, CA stone group, UA stone group, and the mixed CO and CA stone group, while having no impact in the mixed CO stone group and UA stone group. The correlation of Pb with Ca is observed in the CO stone group, UA stone group, and both types of the mixed stone group, though the mechanisms of Pb actions warrant further exploration. Additionally, the substitution effect of Mg, Zn, and Ba on Ca is displayed in the UA stone group and the two types of mixed stone group. There are significant elemental differences among the five types of urinary stones. The assessment of elemental behavior in different types of urinary stones should always take these variations into account. Further studies are warranted to investigate the quantitative analysis of major mixed urinary stones. This will contribute to a more comprehensive understanding of the elemental composition and structural characteristics of mixed stones.

### Electronic supplementary material

Below is the link to the electronic supplementary material.

Supplementary Material 1

## Data Availability

Data is provided within the supplementary information files.
