# Supplementary material for: The key role of major and trace elements in the formation of five common urinary stones
Source: BMC Urol. 2024 May 30;24:114. doi: 10.1186/s12894-024-01498-5 (PMC11138091; doi:10.1186/s12894-024-01498-5)
Supplement: Supplementary file 1 — Supplementary Material 1 [file 12894_2024_1498_MOESM1_ESM.pdf]

Supplementary

The Key Role of Major and Trace Elements in the Formation of Five Common Urinary Stones

Yu Tian [1], Guilin Han [2, 3, *], Shudong Zhang [1, *], Ziyang Ding [2, 3], Rui Qu [2, 3]

[1] Department of Urology, Peking University Third Hospital, Beijing 100191, China; tianyu@bjmu.edu.cn (Y.T.); zhangshudong@bjmu.edu.cn (S.Z.).

[2] Institute of Earth Sciences, China University of Geosciences (Beijing), Beijing 100083, China; dingziyang@email.cugb.edu.cn (Z.D.); qurui@email.cugb.edu.cn (R.Q.).

[3] Frontiers Science Center for Deep-time Digital Earth, Institute of Earth Sciences, China University of Geosciences (Beijing), Beijing 100083, China.

[*] Correspondence: E-mail: hanguilin@cugb.edu.cn (G. H). Tel.: +86-10-8232-3536. Add.: University of Geosciences (Beijing), No. 29 Xueyuan Road, Haidian District.

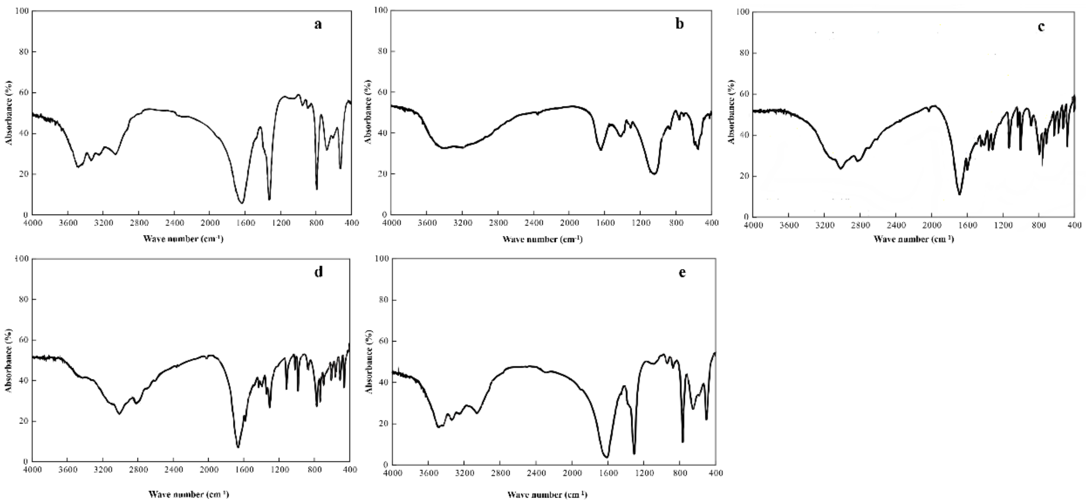

Fig S1 Infrared spectra of typical urinary stones. a. CO; b. CA; c. UA; d. CO+UA; e. CO+CA

Table S1. Major and trace elements of all kidney stones

NO	Gender	Age	Type	Type	Ca	Mg	Na	K	Sr	Zn	Li	Ti	Cu	Se	Rb	Ba	Pb
					%	mg/g	μg/g	μg/g	μg/g	μg/g	μg/g	μg/kg	μg/k	μg/kg	μg/kg	μg/kg	μg/kg
1	Female	54	COM	COD	27.25	3.53	6496.24	903.47	209.65	520.36	21.87	16825.06	8733.04	219.38	211.16	4298.65	7835.61
2	Male	61	COM	COD	26.82	0.40	1937.60	285.20	53.54	28.20	2.30	696.71	641.10	134.51	166.03	485.39	3392.99
3	Female	56	COM	COD	28.11	0.90	2828.01	324.83	141.55	396.65	1.92	2173.31	302.77	198.36	157.08	1821.39	5489.94
4	Female	61	COM	COD	27.23	3.75	3105.27	250.01	130.47	310.63	1.64	2743.71	603.10	105.75	173.34	1345.19	11331.28
5	Male	41	COM	COD	28.69	0.78	1630.16	86.28	98.45	190.22	3.46	1403.41	230.32	49.20	35.80	2424.95	4478.75
6	Male	61	COM	COD	27.84	1.27	3069.87	113.71	109.40	376.53	2.35	2436.32	1530.23	174.32	105.72	1041.56	22120.27
7	Male	60	COM	COD	26.74	0.17	1492.93	202.52	146.22	79.01	5.18	4601.04	203.78	99.67	107.25	246.68	1358.50
8	Male	40	COM	COD	28.40	0.38	1912.44	136.99	96.80	143.73	3.43	2836.75	462.15	190.44	83.92	1371.10	2346.23
9	Male	50	COM	COD	27.30	0.32	1308.19	226.93	67.13	145.26	1.44	718.58	639.40	214.22	162.34	530.85	1233.49
10	Male	41	COM		27.17	0.36	1407.69	262.22	71.90	42.10	3.02	902.48	947.82	244.63	98.24	362.72	1391.50
11	Male	67	COM	COD	27.04	1.32	2909.60	218.16	136.57	628.70	2.03	4635.38	213.55	75.90	129.81	12718.12	7658.03
12	Female	60	COM	COD	25.88	0.43	1662.99	251.71	101.55	20.32	0.84	764.79	565.38	2413.56	122.76	383.83	3502.05
13	Male	68	COM		28.38	1.26	2856.83	117.75	160.97	194.37	2.77	1500.59	329.83	32.90	94.00	1535.00	6391.77
14	Male	57	COM		30.63	3.99	6768.19	226.16	203.34	1230.65	2.92	11452.14	896.24	116.12	105.48	5434.30	13917.92

15	Male	46	COM		29.02	1.25	5238.64	46.32	129.50	693.84	13.04	1560.35	1016.25	225.45	-	1614.31	22451.95
16	Female	61	COM	COD	27.56	2.88	4675.52	161.56	179.36	254.82	2.77	3227.65	388.98	172.29	606.81	7667.19	16008.61
17	Female	69	COM		28.10	1.75	3581.90	264.86	150.69	966.09	3.44	2220.02	271.73	173.23	154.66	2226.47	9533.11
18	Male	72	COM		26.13	0.51	1892.90	253.16	44.86	34.81	1.60	-	582.90	334.99	107.19	51.38	1065.79
19	Male	35	COM		25.19	0.41	881.79	38.02	82.34	510.08	5.23	3360.30	4124.25	160.22	91.89	16385.29	3046.79
20	Male	72	COD	COM	29.61	1.81	5634.30	290.56	304.57	880.96	3.24	3183.20	393.19	299.13	79.69	9388.46	12450.81
21	Male	42	COM		26.63	0.45	1501.67	102.22	71.63	32.47	3.40	846.37	545.10	798.89	97.30	589.65	2104.21
22	Male	40	COM	COD	25.56	1.50	1953.81	284.83	101.63	142.84	1.81	3263.90	279.68	80.90	144.37	3601.90	3052.73
23	Male	77	COM		32.58	10.03	9444.50	553.09	315.45	1633.34	9.19	8508.54	3137.95	115.85	358.35	8421.30	45699.49
24	Male	70	COM		25.43	0.25	1944.65	887.33	104.65	37.93	2.85	627.91	441.21	105.12	46.18	422.54	916.81
25	Male	36	COM	COD	30.59	2.37	5398.65	972.20	170.18	1025.51	5.86	5673.44	791.39	242.58	258.30	5515.89	19385.32
26	Male	28	COM		29.41	4.32	6848.25	1062.91	233.51	1291.18	2.14	10053.14	449.89	202.71	722.40	7495.98	8999.27
27	Male	46	COM		30.25	2.87	3213.17	333.91	160.56	425.32	5.81	741.80	786.35	176.63	114.27	5895.65	8671.10
28	Male	29	COM		32.13	2.08	4820.25	815.89	237.22	1138.97	11.98	5015.93	231.63	199.59	415.33	12579.77	8642.10
29	Male	41	COM		30.92	2.62	4836.51	498.99	252.95	730.89	9.02	38962.76	309.80	118.58	330.86	4475.57	13309.42
30	Female	42	COM		26.33	0.40	1955.85	323.16	59.49	38.61	2.23	908.84	672.42	222.29	149.68	579.54	1963.53
31	Male	52	COM		27.57	1.17	2403.60	116.43	177.78	528.53	2.26	2496.74	253.24	169.56	56.76	6908.06	7064.52
32	Male	48	COM		27.02	0.73	2367.80	54.99	82.87	196.61	2.30	1463.07	337.93	412.31	76.80	587.53	4919.16
33	Female	58	COM	COD	31.74	1.93	6034.44	746.98	139.70	521.35	13.92	28184.73	288.04	279.51	465.66	3370.07	5544.77
34	Male	58	COM	COD	27.69	0.19	1114.42	1.50	51.61	38.52	5.42	1250.92	820.85	280.18	39.27	357.14	2797.26
35	Male	69	COM		26.98	8.25	9231.95	2687.67	401.64	1503.65	2.16	5848.24	1851.92	131.43	2979.65	7906.21	9698.32
36	Male	41	COM	COD	26.68	0.63	1935.63	62.37	115.47	120.20	5.97	524.87	127.61	51.88	117.32	1650.76	2883.68
37	Female	62	COM	COD	27.40	1.49	2519.14	218.96	67.48	149.76	1.77	3053.63	277.41	31.59	126.71	2495.15	2182.80
38	Male	54	COM		27.14	0.28	1677.72	2.26	55.55	19.86	4.68	1114.18	392.38	139.93	61.36	290.65	1530.78
39	Male	73	COM	COD	26.13	0.53	1418.91	210.33	76.15	31.79	3.92	1176.79	633.03	382.37	257.00	416.61	1785.48
40	Male	65	COM		26.56	0.24	1714.49	204.13	68.48	22.96	3.63	1312.46	625.10	138.45	68.74	242.52	1186.66
41	Female	69	COM	COD	26.77	0.73	2382.94	114.83	52.38	189.40	5.96	2765.03	289.65	86.29	110.93	825.81	1772.83
42	Male	68	COM	COD	27.59	1.00	3802.27	250.60	129.38	641.98	1.67	24977.78	347.27	225.25	155.21	1693.12	2752.60
43	Male	60	COM	COD	26.73	0.26	1688.24	173.95	80.52	101.59	1.60	5921.79	275.21	134.74	78.87	456.12	2350.08
44	Male	60	COM	COD	26.84	0.25	1418.84	213.50	69.85	23.00	2.05	5481.46	521.54	337.05	126.67	281.64	1505.88
45	Male	53	COM	COD	25.42	0.62	1431.44	307.19	86.17	50.04	1.73	2631.18	413.85	163.86	201.25	303.97	4248.94
46	Male	79	COM	COD	22.70	29.62	7820.98	679.10	295.63	208.98	2.79	7318.63	264.46	35.95	2205.42	5812.91	6799.01
47	Male	35	COM	COD	27.78	4.58	4701.32	572.28	229.21	494.76	2.63	3633.00	415.26	168.03	419.52	3226.26	5832.82
48	Female	62	COM		25.89	0.29	1433.65	326.77	55.41	16.98	0.98	602.17	400.10	239.35	267.97	271.00	1825.02
49	Male	67	COM		27.97	0.69	2107.55	201.04	66.72	136.64	1.66	2237.90	474.42	184.71	88.92	242.46	7686.83
50	Male	37	COM		28.53	2.78	3679.15	530.88	165.13	742.46	2.38	5590.38	347.82	110.49	220.36	1319.28	12124.13
51	Female	60	CA		21.34	46.62	9349.25	1685.78	455.57	909.62	3.37	4916.98	404.96	55.94	4645.48	4966.84	3036.40
52	Male	73	CA		18.28	44.48	9488.29	1762.03	248.97	668.72	2.95	7282.22	1406.44	36.49	6314.52	4951.72	3343.02
53	Female	50	CA		17.04	51.06	7857.13	1101.80	177.60	569.65	1.90	6329.71	144.54	41.80	3979.58	6152.17	899.24
54	Female	68	CA		18.53	49.70	13554.20	1857.09	211.05	742.33	2.90	4497.71	5731.02	235.04	7587.10	3121.94	2197.47
55	Male	30	CA		18.89	0.52	1436.26	129.13	84.24	285.15	1.75	6872.08	215.12	114.12	76.58	681.01	3247.81
56	Female	60	CA		22.40	35.80	9223.80	1256.40	486.80	1093.90	1093.30	1677.80	1955.00	38.30	3284.80	5409.60	3794.70
57	Male	64	CA		27.40	13.20	727.80	252.90	269.90	900.20	352.90	1658.40	312.20	81.50	2622.00	3599.60	9108.60
58	Male	57	CA		10.40	60.20	6633.70	1171.60	229.00	5168.00	305.60	1750.00	598.20	31.80	7922.50	38978.00	4543.70

59	Male	73	CA		16.30	42.60	9476.00	1745.50	232.10	555.60	773.30	1397.00	1425.70	18.80	5757,3	3465.40	30673.00
60	Male	40	UA		0.65	0.01	414.61	171.97	3.81	1.72	2.54	-	2635.72	465.46	314.40	-	98.76
61	Male	61	UA		0.10	0.05	833.55	221.79	2.69	1.84	2.15	954.93	5031.27	314.76	364.15	1.54	51.47
62	Male	72	UA		7.16	0.20	706.28	188.68	20.10	74.97	2.22	855.15	1734.75	295.26	344.66	1157.01	6537.03
63	Male	43	UA		5.67	0.07	781.76	156.72	22.06	5.77	2.05	-	1185.06	499.40	266.67	93.52	523.12
64	Male	60	UA		0.07	0.03	801.87	258.47	1.83	1.03	1.32	19.74	2025.82	285.79	373.67	14.10	193.18
65	Male	37	UA		5.45	0.09	730.45	226.63	15.65	26.56	2.11	2662.07	1893.85	347.31	251.31	167.79	565.07
66	Male	40	UA		1.40	-	483.80	26.80	2.30	1.30	-	96.50	2813.00	637.20	2275.00	-	211,4
67	Male	58	UA		0.60	-	762.30	14.90	1.30	0.90	-	-	850.00	147.70	3243.00	-	33.70
68	Male	47.00	CA	COM	29.89	3.03	6369.68	496.60	261.68	729.27	2.55	9124.86	399.70	219.74	327.03	6465.94	7397.29
69	Male	45.00	COM	CA	25.36	0.29	1275.38	93.95	63.86	23.26	1.78	622.00	376.52	251.46	79.37	473.73	1907.84
70	Female	77	COM	CA	26.20	0.30	1599.00	-	46.70	129.00	0.10	3954.00	8595.00	1438.00	74.80	1620.90	20458.00
71	Male	47	COM	CA	28.40	2.50	5864.30	2978.00	248.70	616.80	451.20	9920.00	337.70	187.30	1712.00	4536.90	6743.00
72	Male	66	COM	CA	25.40	-	2292.60	-	55.70	12.30	-	-	278.70	98.40	245.10	-	672.10
73	Male	75	COM、COD	UA	6.90	0.06	784.43	259.78	15.79	4.70	2.35	-	1791.47	549.93	369.95	28.33	433.09
74	Male	39	COM、COD	UA	12.92	0.13	1195.20	147.86	33.25	24.22	1.92	501.71	1895.33	247.37	257.49	205.73	1433.44
75	Male	59	COM、COD	UA	8.83	0.09	1737.32	238.45	15.65	8.15	1.65	357.27	2031.40	507.63	336.60	89.76	592.89
76	Male	56	COM	UA	12.80	0.14	724.33	91.92	23.59	15.82	1.38	339.29	2355.01	296.57	267.33	73.86	611.86
77	Male	42	COM、COD	UA	13.11	0.28	1050.42	171.24	39.47	15.45	3.03	124.25	1896.18	224.89	178.55	289.23	714.20
78	Male	53	COM	UA	23.88	0.46	1032.70	159.60	40.59	50.64	1.47	759.92	954.47	436.24	166.61	792.94	829.51
79	Male	50	COM	UA	16.80	0.10	897.00	-	44.50	16.50	-	42.40	1690.70	258.10	143.60	-	15975.00
80	Male	75	COM	UA	6.80	-	818.90	61.90	133.00	3.60	-	52.20	2040.00	443.30	243.20	-	420.00
